# The Pro-197-Thr mutation in the *ALS* gene confers novel resistance patterns to ALS-inhibiting herbicides in *Bromus japonicus* in China

**DOI:** 10.3389/fpls.2024.1348815

**Published:** 2024-02-22

**Authors:** Leicheng Liu, Lamei Wu, Zongfang Li, Yuhang Fang, Boming Ju, Sisi Zhang, Lianyang Bai, Lang Pan

**Affiliations:** ^1^ College of Plant Protection, Hunan Agricultural University, Changsha, China; ^2^ Hunan Agricultural Biotechnology Research Institute, Hunan Academy of Agricultural Sciences, Changsha, China

**Keywords:** acetolactate synthase (ALS), mesosulfuron-methyl, gene mutation, homology modeling and docking, *Bromus japonicus*

## Abstract

**Introduction:**

*Bromus japonicus* is one of the most notorious agricultural weeds in China. The long-term use of ALS-inhibiting herbicides has led to rapid evolution of herbicide resistance in *B. japonicus*. *B. japonicus* population (BJ-R) surviving mesosulfuron-methyl treatment was collected from wheatland. Here, we aimed to confirm the resistance mechanisms in this putative resistant population.

**Methods:**

The dose-reponse tests were used to test the resistance level of the *B. japonicus* to ALS-inhibiting herbicides. Pretreatment with P450 and GST inhibitors and GST activity assays were used to determine whether P450 or GST was involved in the resistance of the BJ-R population. Sanger sequencing was used to analyse the ALS mutation of the BJ-R population. RT-qPCR was used to confirm the the expression levels of the ALS gene in mesosulfuron-methyl -resistant (BJ-R) and-susceptible (BJ-S) *B. japonicus*. An in vitro ALS activity assay was used to determine the ALS activity of the BJ-R and BJ-S populations. Homology modelling and docking were used to determine the binding energy of the BJ-R and BJ-S populations with ALS-inhibiting herbicides.

**Results:**

*B. japonicus* population (BJ-R) was confirmed to be 454- and 2.7-fold resistant to the SU herbicides mesosulfuron-methyl and nicosulfuron, and 7.3-, 2.3-, 1.1- and 10.8-fold resistant to the IMI herbicide imazamox, the TP herbicide penoxsulam, the PTB herbicide pyribenzoxim and the SCT herbicide flucarbazone-sodium, respectively, compared with its susceptible counterpart (BJ-S). Neither a P450 inhibitor nor a GST inhibitor could reverse the level of resistance to mesosulfuron-methyl in BJ-R. In addition, no significant differences in GST activity were found between the BJ-R and BJ-S. *ALS* gene sequencing revealed a Pro-197-Thr mutation in BJ-R, and the gene expression had no significant differences between the BJ-R and BJ-S. The ALS activity of BJ-R was 106-fold more tolerant to mesosulfuron-methyl than that of BJ-S. Molecular docking showed that the binding energy of the ALS active site and mesosulfuron-methyl was changed from -6.67 to -4.57 kcal mol^-1^ due to the mutation at position 197.

**Discussion:**

These results suggested that the Pro-197-Thr mutation was the main reason for the high resistance level of BJ-R to mesosulfuron-methyl. Unlike previous reports of the cross-resistance pattern conferred by this mutation, we firstly documented that the Pro-197-Thr mutation confers broad cross-resistance spectrums to ALS-inhibiting herbicides in *B. japonicus*.

## 1 Introduction

Currently, approximately 160 brome species widely distributed in the temperate and tropical highland areas globally, including 55 species in China (Acedo and Llamas, 2001; Liu et al., 2002). Among these *Bromus* spp. (brome species), the most frequently reported hazardous species are Japanese brome (*Bromus japonicus*), downy brome (*Bromus tectorum*), great brome (*Bromus diandrus*), rigid brome (*Bromus rigidus*), red brome (*Bromus rubens*) and barren brome (*Bromus sterilis*) (Owen et al., 2015; Kumar and Jha, 2017; Johnson et al., 2018; Davies et al., 2020).They primarily infest crops like wheat (*Triticum aestivum*), soybean (*Glycine max*) and oilseed rape (*Brassica rapa*) (Heap, 2003). Brome outbreaks can pose a serious threat to crop yields. For example, the yield of local winter wheat was reduced by 92% when the density of downy brome reached to 538 plants/m^2^ (Rydrych and Muzik, 1968). Brome species have evolved resistance to herbicides with different modes of action, including photosystem II (PSII)-inhibiting herbicides (downy brome in southern Spain) (Menendez et al., 2007), glyphosate (*B. diandrus*, *B. rubens, B. sterilis* in the United Kingdom, Australia and Spain) (Owen et al., 2015; Malone et al., 2016; Johnson et al., 2018; Davies et al., 2020; Vázquez-García et al., 2021), acetolactate synthase (ALS)- and acetyl-coenzyme A carboxylase (*ACCase*)-inhibiting herbicides (*B. diandrus*, *B. rigidurs* in Australia) (Owen et al., 2015). The emergence of herbicide resistance has caused a significant challenge to effective weed control, threatening crop production and food security. More importantly, the fast evolution of herbicide resistance in brome species suggests that herbicide resources available for brome control have been diminishing (Ribeiro et al., 2023).

In recent years, *B. japonicus* and *B. tectorum* have become increasingly damaging and difficult to control in China (Heap, 2003), possibly due to changes in cropping practices and the evolution of herbicide resistance. *B. tectorum* is the main problematic weed in oilseed rape in Qinghai Province (Weng, 2015). When the growth density of *B. tectorum* was 700 plants/m^2^, the yield of local oilseed rape was reduced by 86.6% (Wei et al., 2010). Currently, the herbicides commonly used to control *B. tectorum* in oilseed rape in China are ALS-inhibiting herbicides, mainly including haloxyfop-p-methyl, quizalofop-p-ethyl, and an ACCase-inhibiting herbicide clethodim (Weng, 2015). However, report on evolution of resistance to haloxyfop-p-methyl has been documented in a *B. tectorum* population in Qinghai Province, where the herbicide has been used for many years (Lv et al., 2016). *B. japonicus* has become one of the most troublesome weeds in Hebei and Shandong provinces, the main winter wheat-growing regions in China (Wang et al., 2009; Li et al., 2013). It spreads quickly and causes sharp decreases in crop yields. Prior to 2004, *B. japonicus* was found sporadically only in Shijiazhuang, Hebei Province. However, *B. japonicus* has extremely extended to 62.3 million hm^2^ in Shijiazhuang, with the highest distribution density > 1000 plants/m^2^ after 2009 (Jiang, 2010), leading to wheat yield reduction > 90% (Wei et al., 2010). In 2022, flucarbazone-sodium-resistant *B. japonicus* populations were reported in Tianjin and Hebei (Lan et al., 2022; Li et al., 2022).

Herbicide resistance mechanisms can be divided to two types: resistance conferred by mutations in herbicide target enzyme or target enzyme overexpression (target site resistance [TSR]), and resistance conferred by other mechanisms not involving the target enzyme(non-target site resistance [NTSR]) (Powles and Yu, 2010; Yu and Powles, 2014). To date, studies on the resistance mechanisms of brome species to ALS-inhibiting herbicides have mainly focused on TSR. So far, several target gene mutations have been reported in brome species, including the Pro197-Ser (Park and Mallory-Smith, 2004) (Lan et al., 2022), the Ser653-Asn (Kumar and Jha, 2017), the Trp574-Leu (Davies et al., 2020), and the Asp376-Glu mutations (Li et al., 2022). In addition to TSR, NTSR has also been reported. P450-involved NTSR has been demonstrated in a barren brome population from Czech Republic (Sen et al., 2021) and a *B. japonicus* population from China (Lan et al., 2022).

ALS-inhibiting herbicides are the main herbicides to control *Bromus* spp. in China. These herbicides inhibit the activity of ALS, the first enzyme of chain amino acid biosynthesis, causing leaf necrosis, and finally plant death. Five groups are classified based on their chemical structures: sulfonylureas (SU), imidazolinones (IMI), triazolopyrimidines (TP), pyrimidine thiobenzoates (PTB) and sulfonamide carbonyl triazolinones (SCT) (Tranel and Wright, 2002). In 2021, we collected seeds of surviving *B. japonicus* plants from a wheat field treated with mesosulfuron-methyl in Shandong Province. In this study, we aimed to 1) confirm the resistance level to mesosulfuron-methyl; 2) characterize the cross-resistance patterns and 3) unravel the resistance mechanisms in this putatively resistant population.

## 2 Materials and methods

### 2.1 Plant material

In June 2021, we collected seeds of *B. japonicus* surviving from mesosulfuron-methyl treatment in a wheat field in Yangji District, Jinan, Shandong Province, China. Mesosulfuron-methyl has been used in this wheat field for more than five years. Seeds of the susceptible population (BJ-S) were collected from uncultivated land without herbicide in Xiangfu District, Kafeng, Henan Province, China. Seeds from the two populations were randomly collected from more than 80 field plants and stored in paper bags at 4°C until use.

### 2.2 Herbicides and chemicals

Mesosulfuron methyl [Methyl 2-[(4,6-dimethoxypyrimidin 2-yl)carbamoylsulfamoyl]-4-(methane-sulfonamidomethyl)benzoate] was used for whole-plant dose−response experiments. Five other ALS-inhibiting herbicides (SU nicosulfuron [2-[(4,6-Dimethoxypyrimidin-2-yl)carbamoylsulfamoyl]-N, N-dimethylpyridine-3-carboxamide; IMI imazamox [5-(Methoxymethyl)-2-(4-methyl-5-oxo-4-propan-2-yl-1H-imidazol-2-yl) pyridine-3-carboxylic acid; TP penoxsulam [2-(2,2-Difluoroethoxy)-N-(5,8-dimethoxy-[1,2,4]triazolo[1,5-c]pyrimidin-2-yl)-6-(trifluoromethyl)benzene-sulfonamide; PTB pyribenzoxim [(Benzhydrylideneamino) 2,6-bis[(4,6-dimethoxypyrimidin-2-yl)oxy]benzoate; and SCT Flucarbazone-sodium [Sodium (3 methoxy 4-methyl 5-oxo 1,2,4-triazole 1-carbonyl)-[2-(trifluoromethoxy)phenyl]sulfonylazanide) were used for cross-resistance studies (**Table 1**). Malathion [diethyl 2-dimethoxyphosphinothioylsulfanylbutanedioate, 45% EC, Duyi Jiahe Co., Ltd. Beijing, China] and 4-chloro-nitrobenzoxadiazole (NBD-Cl, 97%) were purchased from Sigma (Sigma Chemical Co.St.Louis, MO, USA) and used as cytochrome P450 monooxygenases (P450) and glutathione S-transferases (GST) inhibitors, respectively.

**Table 1:** Herbicide treatments applied for dose−response to ALS-inhibiting herbicides.

Group	Herbicide	Formulation	Company (city & country)	Dose (g a.i. ha^-1^)	
				BJ-S	BJ-R
SU	Mesosulfuron-methyl	3% OD	FMC (Philadelphia, USA)	1.875, 3.75, 7.5, 15, 30, 60	15,30,60,120,240,480
	Nicosulfuron	40 g L^-1^ OD	JSMONE (Jiangsu, China)	15, 30, 60, 120, 240	30, 60, 120, 240, 480
IMI	Imazamox	4% AS	JSAL (Jiangsu, China)	12, 24, 48, 96, 192	24, 48, 96, 192, 384
TP	Penoxsulam	25 g L^-1^ OD	DOW (Michigan, USA)	7.5, 15, 30, 60, 120	15, 30, 60, 120, 240
PTB	Pyribenzoxim	5% ME	HAILIR (Shandong, China)	9.4, 18.8, 37.5, 75, 150	18.8, 37.5, 75, 150, 300
SCT	Flucarbazone sodium	70% WG	ARYSTA (Shanghai, China)	10.5, 21, 42, 84, 168	21, 42, 84, 168, 336

SU, Sulfonylureas; IMI, Imidazolinone; TP, Triazopyrimidines; PTB, Pyrimidine thiobenzoates; SCT, Sulfonamide carbonyl triazolinones; OD, oil dispersion; AS, aqueous solutions; ME, micro-emulsion; WG, water dispersible granules.

### 2.3 Dose-response to mesosulfuron-methyl and five other ALS-inhibiting herbicides

Seeds from the BJ-R and BJ-S populations were treated with 200 mg L^-1^ GA for 24 h, followed by vernalization at 4°C for 48 h and incubation at 25°C for 72 h in the dark. After seed germination, the seedlings were transplanted to a plastic basin (10 cm in diameter and 12 cm in height). The pots were filled with a 2:1 (w/w) mixture of organic matter and vermiculite, pH 5.6, with 5 seedlings per pot. Seedlings were treated with different doses of mesosulfuron-methyl and other ALS-inhibiting herbicides (**Table 1**) at the 2- to 3-leaf stage using a 3Wp-2000 walking spray tower (Zhongnongjidian) fitted with a 390 ml min^-1^ TP6501E flat fan nozzle delivering 372 L ha^-1^ at a pressure of 3.0 kg cm^−2^. The aboveground tissue of the surviving seedlings were collected, weighed three weeks after treatment (WAT). There were three replicates in each treatment, and the whole experiment was repeated twice during the growing season (October 2022 to April 2023) in a greenhouse (Wang et al., 2021).

### 2.4 Effect of P450 and GST inhibitor pretreatment on mesosulfuron-methyl resistance

The BJ-R and BJ-S seedlings were planted as previously described, followed by treatment with mesosulfuron-methyl in the absence and presence of malathion (a known P450 inhibitor) and 4-chloro-7-nitrobenzoxadiazole (NBD-Cl, a known GST inhibitor) at the 2- to 3-leaf stage, respectively (Wang et al., 2021). Malathion (1000 g a.i. ha^−1^) and NBD-Cl (270 g a.i. ha^−1^) were sprayed 2 and 48 h before the mesosulfuron-methyl treatment, respectively. The spray treatments included mesosulfuron-methyl, malathion, mesosulfuron-methyl and malathion, NBD-Cl, mesosulfuron-methyl and NBD-Cl. The doses of mesosulfuron-methyl were the same as the section 2.3. The fresh weights of surviving seedlings were accessed 3 WAT as described above.

### 2.5 GST activity assay

Seedlings of BJ-R and BJ-S were grown as described above, and treated with 15 g a.i. ha^−1^ mesosulfuron-methyl at the 2-to-3 leaf stage. The aboveground portions from untreated and treated seedlings were collected at 0 hours (CK) and 24 hours for GST activity analysis. The GST activities for BJ-R and BJ-S plants were measured as previously described (Zhao et al., 2019) with minor modifications. Leaf tissue (3 g) was ground to powder in liquid nitrogen. One mL extraction solution (pH=7.5, 0.1 mol L^-1^ Tris-HCl, 2 mmol L^-1^ ethylene diamine tetraacetic acid (EDTA), 1 mmol L^-1^ dithiothreitol (DTT), 0.5% polyvinylpyrrolidone (PVP-40) (w/v)) was added and mixed, then centrifuged at 4°C with 15,000 rpm for 20 min. The supernatant was measured for GST activity at 340 nm in a medium containing 900 µL 0.1 mol L^-1^ K_2_HPO_4_-KH_2_PO_4_ buffer, pH=6.5, 25 µL 40 mM 1-chloro-2, 4-dinitrobenzene (CDNB), and 50 µL 0.1 M reduced glutathione (GSH) in a total volume of 1 ml. Data were measured as the A340 value of the treatment with enzyme extract minus the treatment without enzyme extract (replaced by an equal volume of extraction solution). The whole experiment was conducted twice.

### 2.6 *ALS* gene sequencing and expression

Genomic DNA (gDNA) was extracted from fresh green leaf tissue (100 mg) collected from each plant (10 plants per population) using a Plant DNA Extraction Kit (Tiangen Biotech Co., LTD). The complete *ALS* sequence for *B. japonicus* was amplified using designed primes based on *Bromus tectorum* complete *ALS* sequence (accession MK492423.1). The polymerase chain reaction (PCR) was performed in a volume of 25 μL containing gDNA (2.5 ng), 2× Taq Plus Master MIX II (12.5 μL) (TransGen Biotech), each primer (1 L, 0.1μM), and ddH_2_O. The PCR conditions were as follows: 4 min at 94°C; 35 cycles of 94°C for 30 s, 55°C for 30 s, 72°C for 1 min 30 s; and 7 min at 72°C for final extension. All products were separated on a 1% agarose gel, purified using a gel extraction kit (TransGen Biotech) and sequenced, with sequence data analyzed using BioEdit Sequence Alignment Editor software (Hall, 1999).

Total RNA was isolated from leaf materials from the BJ-R and BJ-S populations using a RNAiso Plus (TaKaRa Biotech, China) kit. Relative expression was conducted with StepOne™ Realtime fluorescence quantitative PCR (RT-qPCR) System (Applied Biosystems) using complementary DNA (cDNA, ~10 ng, synthesized with HiScript II Q RT SuperMix for RT-qPCR (+gDNA wiper), NanJing, China) as templates. The primers were designed as described previously (**Table 2**) (Sen et al., 2021). The expression levels of the *ALS* gene in BJ-R and BJ-S biotypes before and after herbicide treatment were calculated using the 2^-ΔΔCt^ method (Rao et al., 2013; Sen et al., 2021). RT-qPCR experiments were performed with three biological and three technical replicates for each biotype.

**Table 2:** Primers used for *ALS* gene sequencing and RT-Qpcr.

Primers	Sequence (5’-3’)	Annealing temperature (°C)	Usage
ALS-F	ATGGCCACACCCACCACAGCCGCCGTCG	56	Sequencing for *ALS*
ALS-R	TTAATATTCGATCCTGCCATCACCTTC		
UQT-F	GCACAAGCACAAGAAGGTGA	60	The reference gene in RT-qPCR
UQT-R	AGTGGTTTGCCATGAAGGTC		
ALSEXF	ACAGAGTCTGGATTTTGGTCC	60	*ALS* gene in RT-qPCR
ALSEXR	TTATCGCCTCCCCTTTTGTC		

### 2.7 *In vitro* ALS activity assay

The methods for extraction and detection of ALS enzymes were modified from those previously reported (Yu et al., 2010; Fang et al., 2019), and the modification steps are described below. Leaf tissues were collected from plants of each population (1 g), ground to a powder in liquid nitrogen, homogenized in 3× volumes of cold crude enzyme extraction buffer (0.1 mol L^-1^ K_2_HPO_4_-KH_2_PO_4_ buffer, pH=7.5) containing MgCl_2_ (1 mmol L^-1^), thiamine pyrophosphate (TPP, 1 mmol L^-1^), flavin adenine dinucleotide (FAD, 10 μmol L^-1^) and sodium pyruvate (10 mmol L^-1^) to obtain the crude enzyme solution; all the above steps were performed at 0-4 °C. The crude enzyme solution was centrifuged at 4°C at 25,000 g for 15 min, the supernatant was retained, and the same volume of saturated ammonium sulfate solution was added. The enzyme solution precipitated by saturated ammonium sulfate was centrifuged at 4°C at 25,000 g for 15 min, and the precipitate was retained and resuspended in enzyme extraction buffer (4 mL) to obtain the enzyme reaction solution. The enzyme-active reaction system contained enzyme reaction solution (100 µL), enzyme reaction buffer (200 µL, 0.1 mol L^-1^ K_2_HPO_4_-KH_2_PO_4_ buffer pH=7.5) containing MgCl_2_ (20 mmol L^-1^), TPP (2 mmol L^-1^), FAD (20 μmol L^-1^), DTT (1 mmol L^-1^) and sodium pyruvate (200 mmol L^-1^) and different concentrations of mesosulfuron-methyl (100 µL, 0.005, 0.05, 0.5, 5, 50, 500 and 5000 μmol L^-1^). The enzyme-active reaction system was incubated at 37°C for 60 min. 6 N H_2_SO_4_ (40 μL) was used to stop the reaction and incubated at 60°C for 15 min. Finally, creatine solution (0.55%, 190 μL) and alpha-naphthol solution (5.5% in 5N NaOH, 190 μL) were added and incubated at 60°C for 15 min before the enzyme activity was determined by colorimetric assay at 530 nm using commercial acetoin as a standard. Untreated (without herbicide) controls were used as comparisons for each assay system, and each treatment was repeated three times. The entire assay was repeated with independent extracts.

### 2.8 Homology modeling and docking

The spatial structure of the *B. japonicus* ALS protein with proline at position 197 and the ALS protein with threonine at position 197 was reconstructed by homology modeling using the SWISS-MODEL web service (https://swissmodel.expasy.org/). *Arabidopsis thaliana* ALS (PDB ID 7stq.1) (76% identity) was used as a template for the reconstruction of the *B. japonicus* ALS protein (with proline or threonine at position 197) (Fang et al., 2022; Pan et al., 2022). Molecular docking experiments were performed with mesosulfuron-methyl molecules and the ALS-inhibiting herbicides in the ALS active site. The molecular structure of the used ALS-inhibiting herbicides ligand were downloaded from the PubChem website (https://pubchem.ncbi.nlm.nih.gov) and optimized using Chem 3D (Pan et al., 2022). AutoDock Tools-1.5.7 software (Goodsell and Olson, 1990) was used to perform protein-ligand docking between ALS and the herbicide, and the docking result was evaluated via the PLIP website (https://plip-tool.biotec.tu-dresden.de/plip-web/plip/index).

### 2.9 Statistical analyses

Datasets from different replicates of each treatment and experiment were analyzed using SigmaPlot 13.0 (Systat Software, Inc., San Jose, USA). The four-parameter log-logistic curve from SigmaPlot 13.0 was used to determine the rate of herbicide causing 50% growth reduction (GR_50_). The resistance index (RI) was calculated using the proportion of GR_50_ between the BJ-R and BJ-S population. Relative *ALS* expression was compared using *t*-test in Excel (version 2019, Microsoft, Redmond, USA).

## 3 Results

### 3.1 Dose–response to mesosulfuron-methyl and five other ALS-inhibiting herbicides

The dose−response experiments showed that the BJ-R population was more resistant (with GR_50_ value of 605.15 g a.i. ha^-1^) to mesosulfuron-methyl than the BJ-S population (with GR_50_ value of 1.33 g a.i. ha^-1^) (**Table 3**). The BJ-R population was 454-fold more resistant to mesosulfuron-methyl than the BJ-S population.

**Table 3:** Effects of malathion and NBD-Cl on *Bromus japonicus* growth and the response to mesosulfuron-methyl.

Populations	Treatments	GR_50_^a^ (g a.i. ha^-1^) ^b^	RI ^c^
BJ-R	NBD-Cl + Mesosulfuron-methyl	604.45 (10.88)	453.49
	Malathion + Mesosulfuron-methyl	646.40 (4.52)	484.96
	Mesosulfuron-methyl	605.15 (5.20)	454.01
BJ-S	NBD-Cl + Mesosulfuron-methyl	1.38 (0.14)	1.04
	Malathion + Mesosulfuron-methyl	1.53 (0.31)	1.15
	Mesosulfuron-methyl	1.33 (0.17)	–

^a^GR_50_, the herbicide dose causing 50% reduction of fresh weight; ^b^data were shown as mean (SE); ^c^RI, resistance index.

In addition, the BJ-R population was 2.2-, 4.1-, 2.3-, 1.1- and 10.8-fold (**Table 4**) higher for resistance to the SU herbicide nicosulfuron, the IMI herbicide imazamox, the TP herbicide penoxsulam, the PTB herbicide pyribenzoxim and the SCT herbicide flucarbazone, respectively, compared to the BJ-S population. Similar to BJ-S, BJ-R was sensitive to pyraclostrobin and its growth was controlled at the recommended dose. However, the other four ALS-inhibiting herbicides at recommended dose controlled the BJ-R population to a lesser extent than the BJ-S population (**Table 4**). These results indicate that the BJ-R population was highly resistant to the SCT herbicide (RI > 10) and moderately resistant to the SU, IMI and TP herbicides (RI 2-5) and sensitive to the PTB herbicides (RI < 2) (Beckie and Tardif, 2012).

**Table 4:** Dose-response of resistant and susceptible *Bromus japonicus* to ALS-inhibiting herbicides and binding energy of ALS to the herbicides.

Herbicide	Populations	GR_50_ (g ai. ha^-1^) (SE)	RI^a^	Binding energy (kcal mol^-1^)
Nicosulfuron	BJ-R	193.4 (51.2)	2.7 L	-5.32
	BJ-S	70.8 (24.5)	–	-6.44
Imazamox	BJ-R	147.7 (13.6)	7.3 M	-4.85
	BJ-S	20.2 (4.6)	–	-6.33
Penoxsulam	BJ-R	20.40 (1.1)	2.3 L	-2.78
	BJ-S	9.0 (0.8)	–	-4.16
Pyribenzoxim	BJ-R	31.2 (6.2)	1.1 S	–2.82
	BJ-S	29.0 (1.3)	–	-2.86
Flucarbazone sodium	BJ-R	42.1 (15.1)	10.8 H	-2.41
	BJ-S	3.9 (1.3)	–	-4.43

^a^RI, resistance index values were calculated as the ratio between GR_50_ of the resistant and susceptible population; S = not resistant (< 2); L = low resistance (2–5); M = moderate resistance (6–10); H = high resistance (> 10).

### 3.2 Effect of P450 and GST inhibitor pretreatment on mesosulfuron-methyl resistance

**Figure 1:**
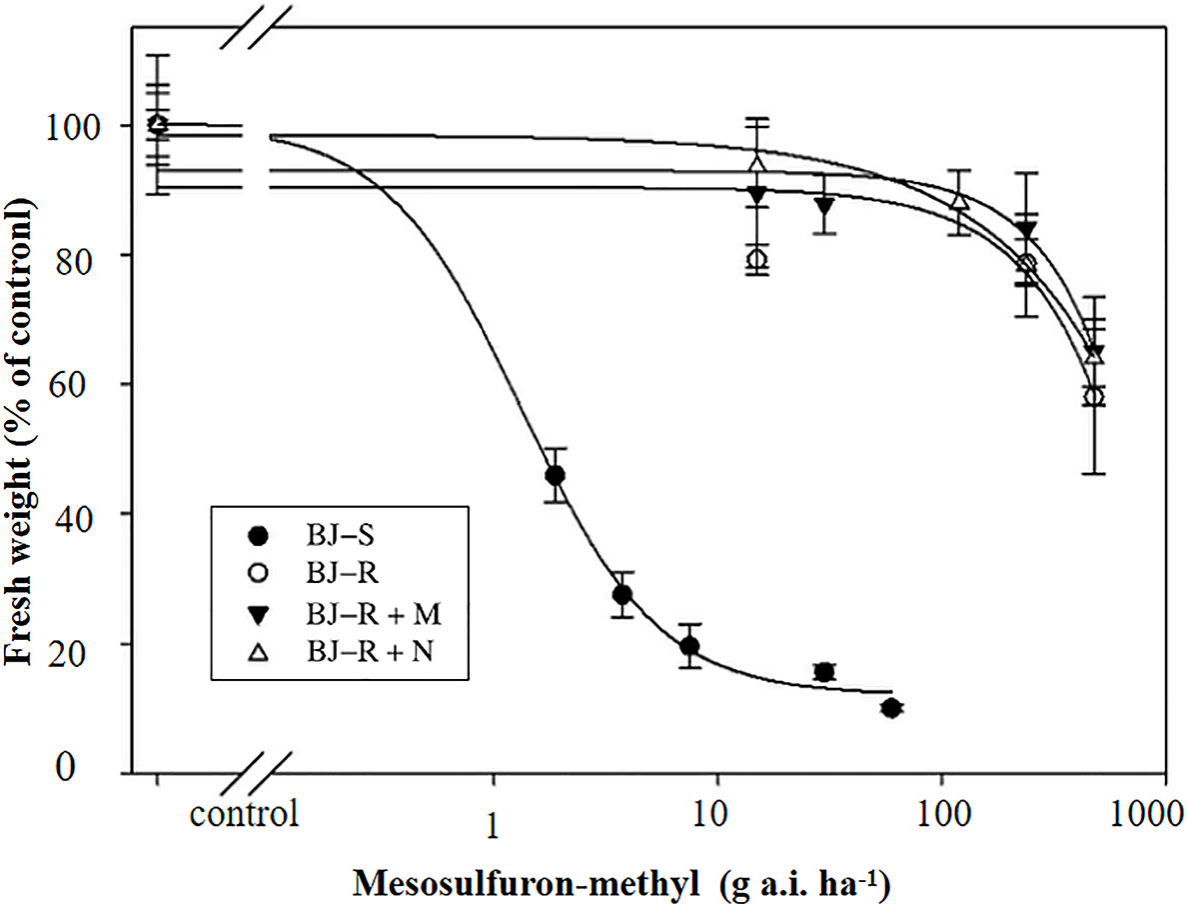
Dose-response of the two *B*. *japonicus* populations to mesosulfuron-methyl, mesosulfuron-methyl plus NBD-Cl, and mesosulfuron-methyl plus malathion. Data are means ± SEM.

Pretreatment with malathion (1000 g a.i. ha^-1^) or NBD-Cl (270 g a.i. ha^-1^) alone had no impact on *B. japonicus* growth. The data showed that the GR_50_ values for pretreatment with malathion or NBD-CI were 646.40 and 604.45 g a.i. ha^-1^, respectively, and were not lower than that with mesosulfuron-methyl alone (GR_50_ value of 605.15 g a.i. ha-^1^) (**Figure 1** and **Table 3**). These results indicated that the resistance of the BJ-R population to mesosulfuron-methyl could not be reversed by malathion or NBD-Cl.

### 3.3 GST activity

**Figure 2:**
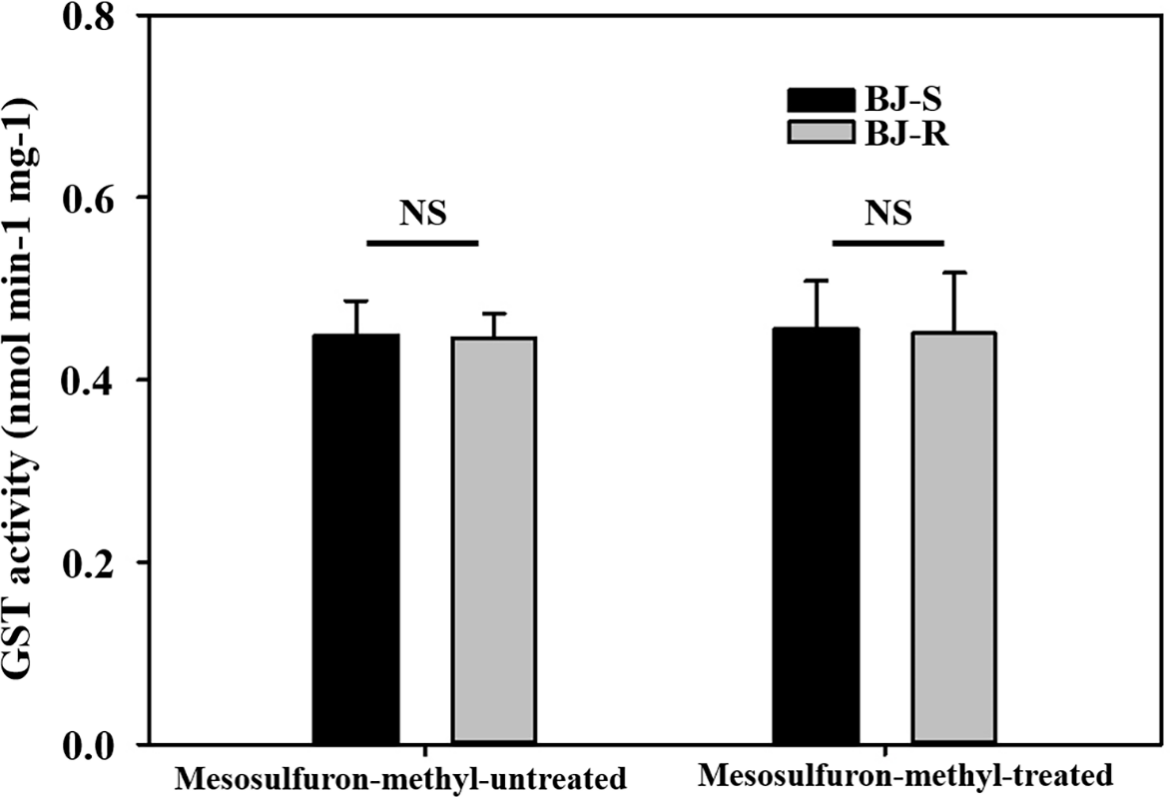
Activities of glutathione S-transferase (GST) with 1-chloro-2,4-dinitrobenzene (CDNB) as a substrate in susceptible (BJ-S) and resistant (BJ-R) plants before and after mesosulfuron-methyl post-treatment. Data are means ± SEM. NS represents not significant at 5% significance level.

To determine whether GST was involved in NTSR to the BJ-R population, the CDNB-GST basal activity of the BJ-R population was 0.985 times higher than that of the BJ-S population when not treated with mesosulfuron-methyl (*P* = 0.889, *t*-test). After mesosulfuron-methyl treatment, the CDNB-GST basal activity of the BJ-R population was 0.989 times higher than that of the BJ-S population (24 h, *P* = 0.927, *t*-test). These results suggest no significant differences in GST activity between the BJ-R and BJ-S populations (**Figure 2**), further excluding the possibility of the involvement of GST in the BJ-R population.

### 3.4 *ALS* gene sequencing and gene expression

**Figure 3:**
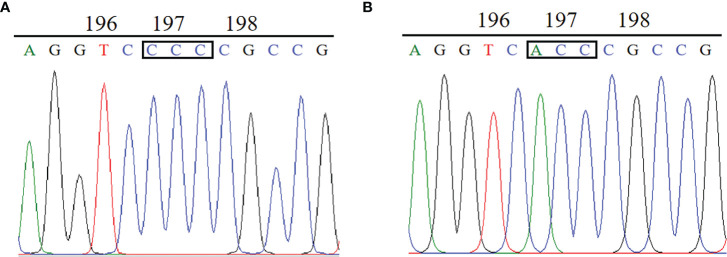
Comparison of ALS gene sequences between the resistant and susceptible plants: **(A)** CCC for Pro in the BJ-S population at codon position 197. **(B)** ACC for Thr in the BJ-R population at codon position 197.

**Figure 4:**
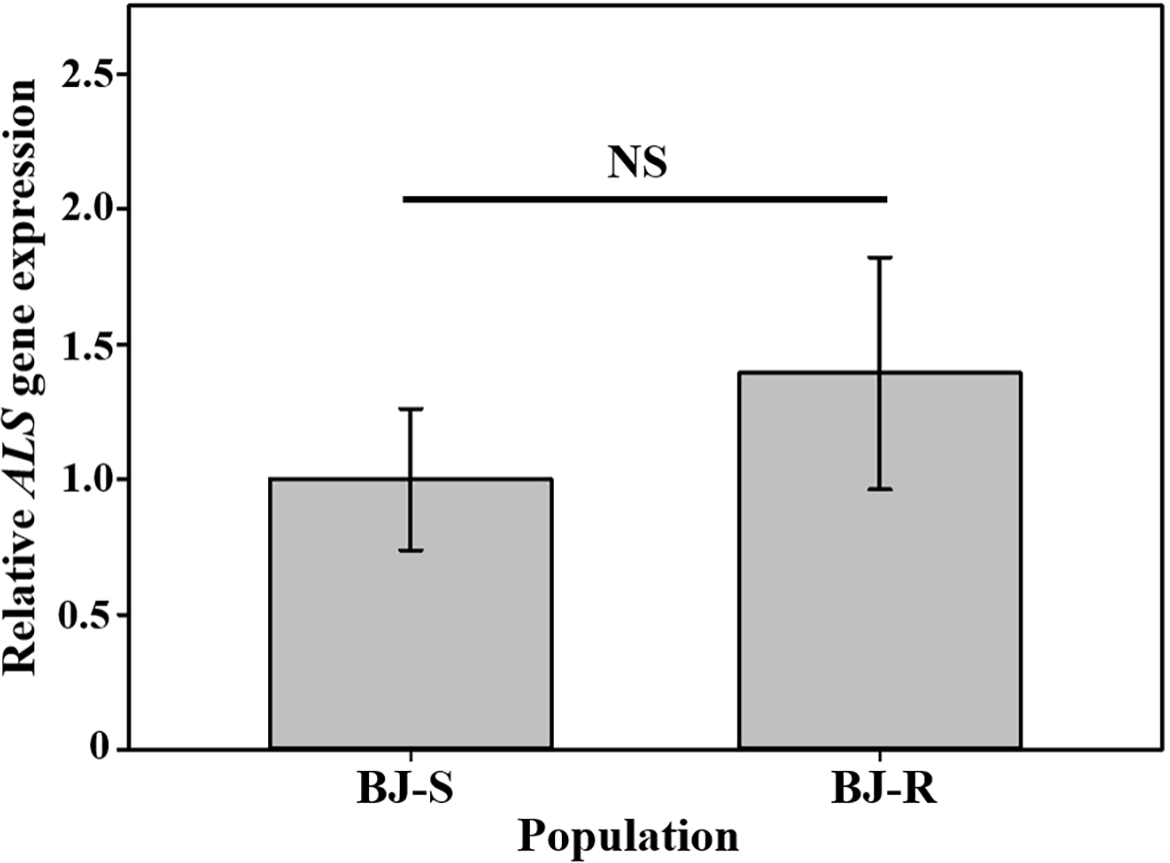
Relative *ALS* gene expression level in BJ-S and BJ-R population after mesosulfuron-methyl treatment. Data are means ± SEM. NS represents not significant at 5% significance level.

The complete *ALS* gene in plants from the BJ-R and BJ-S was amplified and sequenced, covering all reported mutation positions, Ala-122, Pro-197, Ala-205, Phe-206, Asp-376, Arg-377, Trp-574, Ser-653 and Gly-654. The amplified nucleotide sequence was compared with the reported *Bromus tectorum ALS* gene sequence (GenBank accession MK492423.1), and there was 99.8% identity between the BJ-R and BJ-S populations in the nucleotide sequence alignment. Sequencing results showed that codon 197 of the BJ-R samples was substituted from CCC to ACC (**Figure 3**), resulting in a proline to threonine mutation, and all the sequenced samples from BJ-R were consistent. No ALS mutation was detected in the BJ-S samples. The RT-qPCR results showed that the relative expression level of the *ALS* gene in the BJ-R population was 1.39 times (*P* = 0.465, *t*-test) higher than that in the BJ-S population (**Figure 4**), indicating that the *ALS* gene overexpression was not the main reason for the herbicide resistance.

### 3.5 *In vitro* ALS activity assay

**Figure 5:**
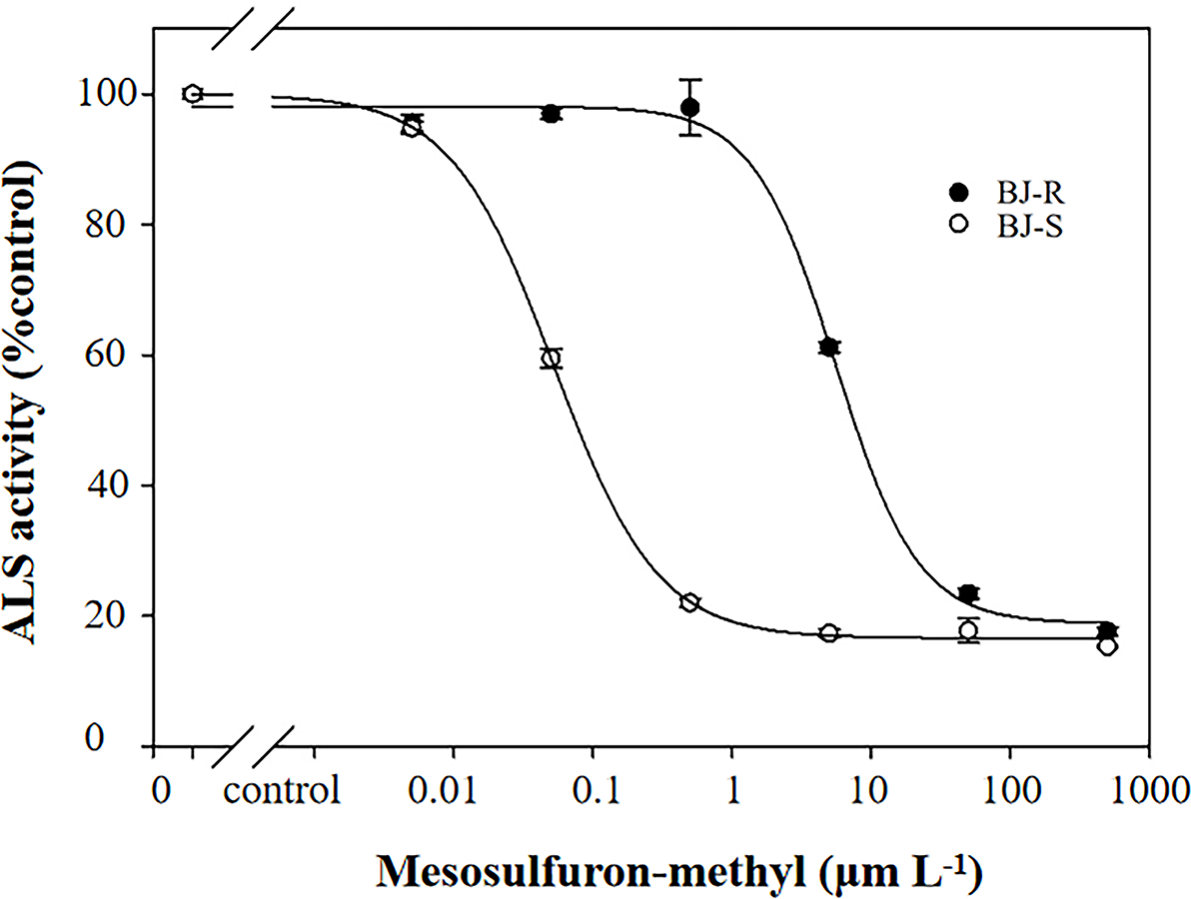
*In vitro* inhibition of ALS activity of the susceptible and resistant populations by mesosulfuron-methyl. Data are means ± SEM.

The inhibitory effect of mesosulfuron-methyl on ALS enzymatic activity was determined using the BJ-R and BJ-S plants. The ALS activity was significantly different between the BJ-R and BJ-S populations after mesosulfuron-methyl treatment (*P* < 0.001, *t*-test, **Figure 5**). The ALS sensitivity *in vitro* to mesosulfuron-methyl was 106.1-fold lower in the BJ-R than in the BJ-S population (**Table 5**). The results showed a trend consistent with the dose-response experiment, further indicating that TSR is the major resistance mechanism in the BJ-R.

**Table 5:** ALS I_50_ values of the resistant and susceptible *Bromus japonicus* populations for mesosulfuron-methyl.

Population	I_50_^a^ (µm L^-1^) (SE)	RI ^b^
BJ-R	5.57 (0.60)	106.1
BJ-S	0.053 (0.003)	–

^a^I_50_, the dose of herbicide required to inhibit ALS activity by 50%; ^b^RI, resistance index values were calculated as the ratio between I_50_ of the resistant and susceptible population.

### 3.6 Homology modeling and docking

**Figure 6:**
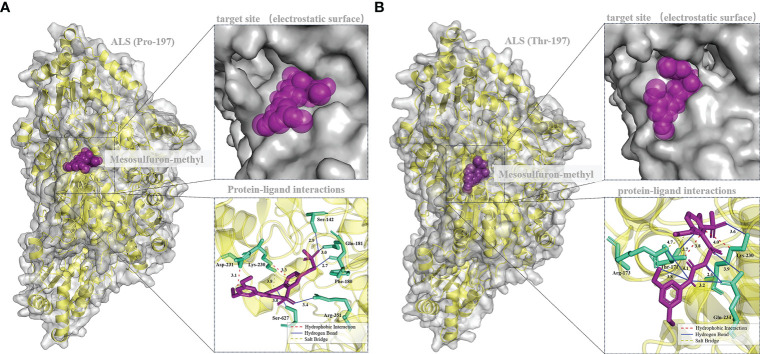
Molecular docking conformation of mesosulfuron-methyl and the ALS protein. **(A)** Docking conformation of mesosulfuron-methyl and the BJ-S (Pro-197) ALS protein, Ser-142 equivalent to Ser-168 in *B*. *japonicus* ALS, Phe-180 equivalent to Phe-206 in *B*. *japonicus* ALS, Gln-181 equivalent to Gln-207 in *B*. *japonicus* ALS, Lys-230 equivalent to Lys-256 in *B*. *japonicus* ALS, Asp-231 equivalent to Asp-257 in *B*. *japonicus* ALS, Arg-351 equivalent to Arg-377 in *B*. *japonicus* ALS, Ser-627 equivalent to Ser-653 in *B*. *japonicus* ALS. **(B)** Docking conformation of mesosulfuron-methyl and the BJ-R (Thr-197) ALS protein, Thr-171 equivalent to Thr-197 in *B*. *japonicus* ALS, Arg-173 equivalent to Arg-199 in *B*. *japonicus* ALS, Lys-230 equivalent to Lys-256 in *B*. *japonicus* ALS, Gln-234 equivalent to Gln-260 in *B*. *japonicus* ALS.

**Figure 7:**
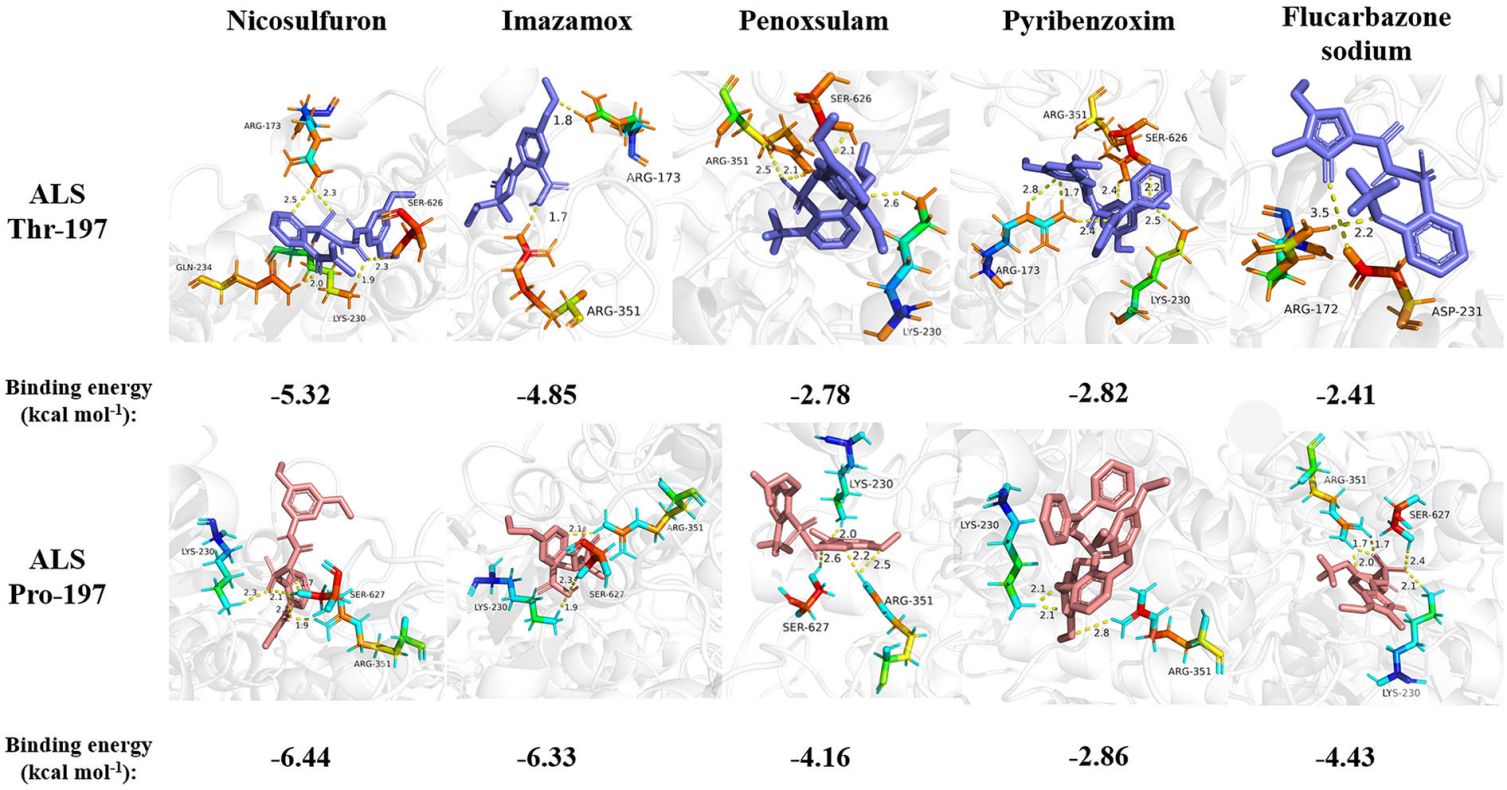
Docking conformation of the BJ-R (Thr-197) and BJ-S (Pro-197) ALS protein to nicosulfuron, imazamox, penoxsulam, pyribenzoxim, flucarbazone sodium, respectively. Dotted yellow line: hydrogen bond. The number next to the yellow dashed line represents the hydrogen bond length. The number at the bottom of the docking result picture represents its binding energy.

Mesosulfuron-methyl was docked into the active site cavity of the reconstructed 3D ALS using AutoDock Tools-1.5.7 software (**Figure 6**). The binding site area of the wild-type (BJ-S) active site cavity is larger than that of the mutant type (BJ-R), which may be caused by the binding site cavity squeezing by the Arg173 and Glu234 in the mutant type. Three types of interactions (salt bridge, hydrogen bond and hydrophobic interaction) were detected in the mutant and wild type docking models; the average distances of hydrophobic interactions, hydrogen bonds and salt bridges in the wild type were 3.2, 3.0 and 3.8 Å, respectively, shorter than those in the mutant type (the average distances of the same types of interactions were 3.9, 3.6 and 4.7 Å, respectively). Furthermore, the Pro197-Thr mutation further narrowed the active site channel of herbicide binding to the ALS protein (**Figure 6**), indicating that the wild type is more easily to dock with mesosulfuron-methyl. In addition, the free interaction (binding) energy between the mesosulfuron-methyl and the wild type was -6.67 kcal mol^-1^ and that of the mutant type was -4.57 kcal mol^-1^, indicating that the wild type has a stronger binding ability to mesosulfuron-methyl than the mutant type. In addition, the binding energies of the wild type and mutant type ALS proteins to other ALS-inhibiting herbicides were shown in **Table 4** and **Figure 7**, and the differences between the changes of binding energy are consistent with the results of the cross-resistance patterns in the BJ-R.

## 4 Discussion

In this study, we found a *B. japonicus* population, BJ-R, was highly resistant to mesosulfuron-methyl conferred by the Pro-197-Thr mutation. To our knowledge, this is the first report on the Pro-197-Thr mutation identified in a brome species. The Pro-197-Thr mutation has previously been reported in a variety of weeds, but the mechanisms and levels of resistance conferred by this mutation vary. In particular, we found that the Pro-197-Thr mutation confers cross-resistance to IMI herbicides in *B. japonicus*, but still sensitive to PTB herbicides. This cross-resistance patterns are different from those reported in literatures (**Table 6**). According to Kaloumenos et al., a population of corn poppy (*Papaver rhoeas*) containing the Pro-197-Thr mutation was identified to be resistant to IMI herbicide (Kaloumenos et al., 2011). However, different from our study (**Table 4**), corn poppy carrying this mutation evolved resistance to a PTB herbicide, pyrithiobac. In addition, Délye et al. identified a population of poppy plants carrying the Pro-197-Thr mutation exhibited a resistance pattern similar to that observed by Kaloumenos et al. (Délye et al., 2011; Kaloumenos et al., 2011). However, it is noteworthy that most of their mutant plants were susceptible to florasulam, and only four homozygous mutants conferred resistance to this herbicide (Délye et al., 2011). *Chrysanthemum coronarium* population carrying the Pro-197-Thr was found to be resistant to SU, TP, SCT, PTB and IMI herbicides (Tranel et al., 2003). This resistance pattern closely resembles the broad cross-resistance pattern proposed by Scarabel et al. (Tranel et al., 2003; Scarabel et al., 2004). In addition, a *Kochia scoparia* population containing the Pro-197-Thr mutation was identified as susceptible to imazethapyr (Guttieri et al., 1992), and a wild radish population carrying this mutation was also susceptible to IMI herbicides (Yu et al., 2012). In addition, Sada et al. found a *Schoenoplectiella juncoides* population with the Pro-197-Thr mutation that was susceptible to imazaquin-ammonium (Sada et al., 2013).

**Table 6:** Cross-resistance patterns conferred by the Pro-197-Thr mutations in herbicide-resistant weeds. .

Amino aicd mutation	Species	ALS-inhibiting herbicides					Reference
		PTB	IMI	SCT	SU	TP	
Pro 197 Thr	*Kochia scoparia*	ND	S	ND	R	R	(Saari et al., 1990; Guttieri et al., 1992, 1995)
	*Raphanus raphanistrum*	ND	S	ND	R	R	(Tan and Medd, 2002; Yu et al., 2003, 2012)
	*Papaver rhoeas*	r	r	ND	R	r	(Scarabel et al., 2004; Kaloumenos et al., 2009; Délye et al., 2011; Kaloumenos et al., 2011)
	*Chrysanthemum coronarium*	R	r	R	R	r	(Tranel et al., 2003)
	*Lactuca serriola*	ND	r	ND	R	r	(Preston et al., 2006)
	*Alopecurus myosuroides*	ND	ND	ND	R	ND	(Délye and Boucansaud, 2008)
	*Descurainia sophia*	ND	ND	ND	R	ND	(Cui et al., 2008; Cui et al., 2011b)
	*Apera spica-venti*	ND	ND	r	R	r	(Krysiak et al., 2011; Massa et al., 2011; Hamouzova et al., 2014)
	*Anthemis cotula*	ND	r	ND	R	r	(Intanon et al., 2017)
	*Capsella bursa-pastoris*	ND	ND	ND	R	ND	(Cui et al., 2011a)
	*Hordeum murinum ssp. leporinum*	ND	r	ND	R	ND	(Owen et al., 2012b)
	*Schoenoplectus juncoides*	ND	S	ND	R	ND	(Sada et al., 2013)
	*Alopecurus aequalis*	ND	r	ND	r	r	(Xia et al., 2015)
	*Galium aparine*	ND	ND	ND	R	ND	(Zhang et al., 2016)
	*Bromus japonicus*	S	r	R	r-R	r	

This table was cited from www.weedscience.org, S = Susceptible biotype, r = Moderate resistance (< 10-fold relative to sensitive biotype), R = High Resistance (> 10-fold), ND = Not Determined.

Resistance patterns to ALS-inhibiting herbicides conferred by the same mutation mainly depend on several reasons. First, weed species. It is believed that cross-resistance patterns are more closely related to species than to “standard” types, which is also not difficult to see from the **Table 6** (Tranel and Wright, 2002; Tranel et al., 2003). Second homo/heterozygotes of target gene? However, the resistance factor of the Th-03F population carrying the heterozygous Pro-197-Thr mutation was higher than 2400, whereas that of the homozygous mutant K-10F was only 1860 (Kaloumenos et al., 2011), combined with the experimental results of Délye et al. (only corn poppy populations carrying the homozygous Pro-197-Thr mutation were able to develop resistance to florasulam) (Délye et al., 2011), it is not difficult to conclude that plant heterozygotes and homozygotes can greatly influence the level and mode of herbicide resistance in plants. Third, different herbicide groups? Yu et al. argues that the same mutant form will have different levels of resistance to different members of the same class of herbicide (Yu et al., 2012), which was also supported by our study. The resistance levels to mesosulfuron-methyl and nicosulfuron by the BJ-R population were 454 and 2.7, respectively. It is hypothesized that these weed species containing the Pro-197-Thr mutation may also have other coexisting resistance mechanisms, including *ALS* gene overexpression or enhanced metabolism (Gaines et al., 2020; Lan et al., 2022). The role of *ALS* gene expression in the development of resistance is relatively complex. Some studies have found that *ALS* gene expression in resistant populations is higher than that in sensitive populations (Sen et al., 2021), but some studies have shown that *ALS* gene expression in resistant populations is lower than or equal to that in sensitive populations (Yu et al., 2003; Fang et al., 2022). In this study, we have ruled out the existence of *ALS* gene overexpression in the BJ-R population (**Figure 4**).

Increased metabolic capacity can reduce herbicide damage to weeds and lead to changes in weed resistance patterns and levels (Powles and Yu, 2010). Davies et al. found that the P450 gene may contribute to resistance to mesosulfuron and pyroxsulam in *Bromus sterilis* (Davies et al., 2020). Sen et al. also found that the P450 gene may work together with *ALS* gene overexpression to cause resistance to pyroxsulam in *Bromus sterilis* (Sen et al., 2021). Owen et al. discovered that the P450 gene is responsible for sulfosulfuron resistance in *Bromus rigidus* (Owen et al., 2012a), and Park et al. demonstrated that the P450 gene accelerates metabolism in *Bromus tectorum*, leading to resistance to propoxycarbazone-sodium (Park et al., 2004). In particular, Lan et al. found that the Pro-197-Ser mutation and P450 together mediate resistance to fluzoxuron in B.*japonicus* (Lan et al., 2022). Unfortunately, they did not perform a wide range of cross-resistance experiments, so we could not obtain information on the influence of metabolic enzymes on cross-resistance. To further investigate whether metabolic enzymes were involved in the resistance of the BJ-R population to mesosulfuron-methyl, the BJ-R and BJ-S populations were treated with the P450 inhibitor malathion and the GST inhibitor NBD-Cl in combination with mesosulfuron-methyl. The results showed that neither malathion nor NBD-Cl reversed resistance to mesosulfuron-methyl in the BJ-R population, demonstrating that resistance to mesosulfuron-methyl in the BJ-R population may not be related to enhanced metabolism induced by P450 and GST. GST activity assay further confirmed that GST was found to be not involved in mesosulfuron-methyl resistance of the BJ-R population (**Figure 2**). Because *B. japonicus* seedlings cannot survive in dark conditions, the P450 activity assay was not performed as we could not collect microsomes from them assay (Wang et al., 2013). Therefore, we cannot completely rule out the involvement of P450 in mesosulfuron-methyl-resistant BJ-R population. However, in addition to P450 and GST, there are metabolic enzyme gene families such as GT, ABC and AKR that can alter herbicide penetration into the plant, rates of herbicide translocation and rates of herbicide sequestration metabolism (Zhao et al., 2018; Pan et al., 2019), such mechanisms act to minimize the amount of herbicide reaching the target site and lead to changes in the level of plant resistance levels (Powles and Yu, 2010). Therefore, without more detailed herbicide metabolism and uptake experiments, we cannot completely rule out the involvement of these metabolic gene families, and they may also be one reason for the different cross-resistance patterns. From the above speculation, it is not difficult to see that there are many factors that influence the resistance pattern and resistance level of plants. It is very important that we be precise to a specific species to understand its resistance mechanism and to implement more precise and effective prevention and control measures against it.

The *in vitro* ALS enzyme activity of the BJ-R population was less sensitive to mesosulfuron-methyl than that of the BJ-S population, indicating that the resistance of BJ-R population was related to alterations in ALS activity. Saari et al. found that a population of *Kochia scoparia* was resistant to sulfonylurea herbicides, having ruled out *ALS* expression and plant uptake and metabolism of herbicides, they hypothesized that the resistant biotype was resistant to sulfonylurea simply because its ALS enzyme was not sensitive to sulfonylurea herbicides (Saari et al., 1990). Unfortunately, they did not test for *ALS* gene target mutations in this biotype, as changes in ALS enzyme activity are usually accompanied by *ALS* gene target mutations. In addition, many studies have reported that target mutations reduce the sensitivity of ALS enzyme activity to herbicides, thus making plants resistant to herbicides (Fang et al., 2022; Lan et al., 2022; Li et al., 2022). In general, the sensitivity of ALS enzyme activity to herbicides is usually negatively correlated with plant resistance to herbicides, i.e., the ALS mutant resistant population has a lower sensitivity to herbicides than the sensitive populations (Yu et al., 2010), which is also consistent with our study.

It has been reported that any mutation in the Pro197 residue confers resistance to SUs (McCourt et al., 2006). This is because Pro197 is located at one end of a ɑ-helix at the entrance to the active site access channel of the ALS protein channel. This residue is directly contact with the aromatic ring of SU, and the contact is easily affected by the substitution in the Pro197. Changes in the Pro197 causes alterations of spatial structure of the protein, leading to disruption of the contact (McCourt et al., 2006). Combining this finding with our results, we suggest that the BJ-R resistance to mesosulfuron-methyl is due to the Pro197 substitution, reducing the binding affinity of ALS to the herbicides.

The long-term use of herbicide with single site of action have inevitably promoted the evolution of herbicide resistance in weeds. In this study, long-term use of mesosulfuron-methyl has resulted in the local *B. japonicus* population (BJ-R) to become resistant to most ALS-inhibiting herbicides. Increasing the rate of mesosulfuron-methyl or simply replacing with another ALS-inhibiting herbicide is not an effective way for controlling this population. Therefore, it is necessary to change other herbicides with different sites of action, or apply integrated weed control measurements to reduce the overreliance on chemical herbicides. It is important to use chemical herbicides scientifically to slow down the evolution of herbicide resistance in weeds, and to protect limited herbicide resources by taking measurements as described above.

## 5 Conclusion

In conclusion, we identified the Pro-197-Thr mutation in *B. japonicus* for the first time, elucidated the resistance mechanism to mesosulfuron-methyl and characterized the cross-resistance pattern in the BJ-R population. BJ-R population is still susceptible to the PTB herbicide pyribenzoxim, and this class of herbicide is likely to be still effective in *B. japonicus*.

## Data availability statement

The data presented in the study are deposited in the GenBank repository, accession numbers PRJNA1048870 and SRR27070961 for wild type and mutant ALS genes in *B. japonicus* respectively.

## Author contributions

LL: Conceptualization, Data curation, Formal analysis, Methodology, Writing – original draft, Writing – review & editing. LW: Conceptualization, Data curation, Formal analysis, Funding acquisition, Methodology, Writing – original draft. ZL: Conceptualization, Data curation, Writing – original draft. YF: Data curation, Methodology, Writing – original draft. BJ: Formal analysis, Methodology, Writing – original draft. SZ: Formal analysis, Methodology, Writing – original draft. LB: Formal analysis, Funding acquisition, Writing – original draft. LP: Data curation, Formal analysis, Funding acquisition, Methodology, Project administration, Writing – original draft, Writing – review & editing.
